# Machine learning for predicting lifespan-extending chemical compounds

**DOI:** 10.18632/aging.101264

**Published:** 2017-07-18

**Authors:** Diogo G. Barardo, Danielle Newby, Daniel Thornton, Taravat Ghafourian, João Pedro de Magalhães, Alex A. Freitas

**Affiliations:** ^1^ Integrative Genomics of Ageing Group, Institute of Ageing and Chronic Disease, University of Liverpool, Liverpool, UK; ^2^ Department of Psychiatry, University of Oxford, Warneford Hospital, Oxford, UK; ^3^ School of Life Sciences, University of Sussex, Falmer, Brighton, UK; ^4^ School of Computing, University of Kent, Canterbury, UK

**Keywords:** longevity, anti-ageing drugs, pharmaceutical interventions, ageing, bioinformatics, *C. elegans*, machine learning

## Abstract

Increasing age is a risk factor for many diseases; therefore developing pharmacological interventions that slow down ageing and consequently postpone the onset of many age-related diseases is highly desirable. In this work we analyse data from the DrugAge database, which contains chemical compounds and their effect on the lifespan of model organisms. Predictive models were built using the machine learning method random forests to predict whether or not a chemical compound will increase *Caenorhabditis elegans*’ lifespan, using as features Gene Ontology (GO) terms annotated for proteins targeted by the compounds and chemical descriptors calculated from each compound's chemical structure. The model with the best predictive accuracy used both biological and chemical features, achieving a prediction accuracy of 80%. The top 20 most important GO terms include those related to mitochondrial processes, to enzymatic and immunological processes, and terms related to metabolic and transport processes. We applied our best model to predict compounds which are more likely to increase *C. elegans*’ lifespan in the DGIdb database, where the effect of the compounds on an organism's lifespan is unknown. The top hit compounds can be broadly divided into four groups: compounds affecting mitochondria, compounds for cancer treatment, anti-inflammatories, and compounds for gonadotropin-releasing hormone therapies.

## INTRODUCTION

Old age is the greatest risk factor for many diseases, including various types of cancer, inflammatory and neurodegenerative diseases. Traditional medical science combats one disease at a time, instead of combating the underlying biological ageing process that leads to many age-related diseases. From a whole body system's point of view, this traditional one-disease-at-a-time approach focuses on the downstream diseases, rather than considering the underlying mechanisms of age-related functional decline. This approach has limited effectiveness at present and is likely to be less effective in the future, because of an increasingly larger elderly population suffering from multiple age-related diseases. In contrast, interventions that slow down ageing and promote “healthy ageing” could in principle delay the onset of all age-related diseases, with a significant benefit to human health and a large reduction of healthcare costs [1].

Pharmacological interventions are arguably the most practical ageing intervention for humans, avoiding the main problems with genetic interventions (generally unethical in humans) and dietary interventions such as caloric restriction, which are difficult to maintain for the vast majority of people. For instance, there is currently great interest in discovering drugs that mimic the process of caloric restriction (caloric restriction mimetics) [2,3]. In addition, promising research on pharmacological interventions on the ageing process is underway at the National Institute of Aging's Intervention Testing Program (ITP), which consists of administering drugs or chemical compounds to mice under carefully controlled conditions [4,5]. However, as mouse experiments are costly and time consuming, so far only a limited number of drugs or compounds have been evaluated. Thus, using simpler model organisms for evaluating a chemical compound's effect on an organism's lifespan is appealing, and a substantially larger number of studies have administered compounds to *C. elegans* than other organisms. As the ITP for mice, the *Caenorhabditis* Intervention Testing Program has been introduced for assessing longevity variation for chemical compounds [6]. Although *C. elegans* is physiologically different from humans, *C. elegans* is the most studied model organism in ageing research, producing insights that are applicable to other organisms [7], since cellular-level ageing processes are often conserved across distantly-related species [8]. According to the GenAge database [9], *C. elegans* is the animal model with by far the most known ageing-related genes (838 at the time of writing).

In this work we analyse data from the DrugAge database [10], which contains information about chemical compounds and their effect on the lifespan of organisms. DrugAge contains a variety of compounds with anti-ageing properties such as gerosuppressant, geroprotective and senolytic activity [11–13] as well lifespan increasing properties for a specific species. Existing databases with lifespan-extending drugs include AgeFactDB (http://agefactdb.jenage.de/) [14], and Geroprotectors.org [15] (http://geroprotectors.org/). DrugAge incorporates data from these resources and improves on them by providing a more extensive and systematic repertoire of lifespan-extending drugs, compounds and substances. DrugAge is manually curated and features only information relative to lifespan assays conducted in well-controlled studies. DrugAge contains data about several model organisms, and the majority of compounds in DrugAge have been evaluated on *C. elegans*, so we focus on analysing data for this organism.

In order to analyse such data, we use random forests, which is a supervised machine learning method – for a recent review of supervised machine learning applied to the biology of ageing, see [16]. In this work, the random forest builds a classification model to predict whether or not a chemical compound will increase the lifespan of *C. elegans*, based on predictive features describing that compound. We created datasets with two types of predictive features, namely Gene Ontology (GO) terms annotated for proteins interacting with the compounds and chemical descriptors calculated from each compound's chemical structure. In order to evaluate the predictive relevance of these two types of features, we created three different datasets: one using as predictive features only the GO terms, another using as predictive features only the chemical descriptors, and a third dataset using both types of features. In addition, the best model produced by the random the forest method was applied to a screening “external” dataset with com-pounds from the DGIdb database, where the effect of the compounds on an organism's lifespan is unknown. The predictions of that model were used to identify the “top hit” compounds in the DGIdb dataset, i.e. compounds with higher probabilities of increasing lifespan in *C. elegans*.

There are some related works that performed data analysis on compounds increasing *C. elegans*’ lifespan, but without using any predictive machine learning method. In particular, Ziehm et al. used an empirical scoring function combining several different factors to evaluate the relevance of a compound for ageing [17]; and Ye et al. (2014) constructed a pharmacological network in order to reveal pharmacological classes most related to *C. elegans*’ ageing [18]. In addition, Calvert et al. 2016 identified drugs which induce gene expression profiles similar to the profiles of genes associated with caloric restriction (CR), and observed that various genes targeted by lifespan-extending drugs are included in CR and longevity networks [3]. Furthermore, Aliper et al. [19] utilised computational tools to carry out signalling pathway analysis of gene expression between young and old stem cells in humans. Based on the signalling pathway results, known compounds were screened and ranked, in order to identify the best compounds to target those pathways and restore a “young” cellular profile. A review of several specific pharmacological classes extending *C. elegans*’ lifespan can be found in Carretero et al. 2015 [20], but again with no use of predictive machine learning methods.

To the best of our knowledge, this is the first work to propose the use of a predictive machine learning method (namely Random Forests) to analyse data about the effect of chemical compounds in C. elegans’ lifespan, as well as the first work to apply machine learning to data about compounds in the DrugAge database.

## RESULTS AND DISCUSSION

### Predictive accuracy of the models

We have created a DrugAge dataset specifically for studying the classification of compounds into the classes “increase lifespan” or “do not increase lifespan”, depending on each compound's effect when administer-ed to *C. elegans*. In this dataset, each compound to be classified belongs to one of the two just-mentioned classes, and is described by a large set of chemical descriptors and biological GO term features.

We use the random forest method as the classification algorithm to analyse this dataset. This type of method was chosen because it is particularly popular in bioinformatics [21,22], it is robust to overfitting in datasets where the number of features is much larger than the number of instances (as with our dataset) [22,23], it is relatively simple to understand and to use, and finally, in contrast to other state-of-the-art classification methods like support vector machines, random forests produce interpretable results based on a variable (feature) importance measure, an interpretation mechanism also exploited in this paper.

Predictive accuracy for the models developed was evaluated by Area Under the ROC curve (AUC). This is a measure between 0 and 1, with 1 indicating perfect (no error) class predictions. The reported predictive accuracy used is the median over the 10 test sets of the external cross-validation. We report the median accuracy, rather than the mean, because the former is more robust to outliers. The median AUC results from each of the different versions of the DrugAge dataset (using either chemical and/or biological descriptors), where for each dataset version we optimised the parameters ntrees and mtry of the random forest method as described in the Methods section.

The AUC results are reported in Table 1. Comparing the AUC values across the dataset versions (last column in Table 1), it is clear that, in general, the set of chemical descriptors have a greater ability to predict a compound's class than the set of GO terms. More precisely, the dataset using only chemical descriptors as features has substantially larger AUC than the one using only GO terms as features (0.781 vs. 0.716, respectively). However, the GO term features still offer some positive contribution to the predictive accuracy of random forests, since the dataset version leading to the highest AUC value in Table 1 (0.800) was the one using both GO terms and chemical descriptors as features.

**Table 1:** Predictive accuracy (median AUC values on 10-fold cross validation) obtained by random forest with parameters optimized for each DrugAge dataset version (each with a different feature type combination)

Dataset features	RF's optimized parameters		Median AUC
	ntrees	mtry	
GO terms only	300	52	0.716
Chemical descriptors only	100	16	0.781
GO terms and chemical descriptors	900	210	0.800

### Biological and chemical features for the prediction of longevity compounds in *C. elegans*

One of the benefits of utilizing the random forest method, as well as it being a highly predictive technique, is that for each feature an importance measure can be calculated. This importance measure (often called variable importance) offers the opportunity to interpret the relevance of each feature in the model produced. In this work, using the Boruta and Ranger R packages [21,24] and computing the importance of features in the best model (built using both GO terms and chemical descriptors as features), 93 features – 73 chemical descriptors and 20 GO terms – were selected as statistically significant features (full table Supplemental Data). Recall that the GO term features are derived from the proteins which are targeted by each compound.

The 20 GO terms selected as significant mainly make up biological process GO terms (14 out of 20), five molecular function terms and one defining a cellular component term. Biological process GO terms describe a series of processes as well as specific biological processes such as macromitophagy and macroautophagy, which are among the features with the highest importance in this work. Molecular function GO terms describe specific activities that occur at the molecular level such as isomerase activity and protein disulfide isomerase activity. Finally, cellular component GO terms describe locations in the cell, e.g. at the level of organelles or macromolecular complexes such as the mitochondrial proton-transporting ATP synthase complex, highlighted as the only significant cellular component GO term feature in this work.

Chemical molecular descriptors are calculated from the chemical structure and are normally used to build predictive models to study the relationship between a compound's chemical structure and its biological and pharmacokinetic properties such as drug distribution and absorption [25,26]. This paper is the first use of chemical molecular descriptors (as well as GO terms) to study the relationship between longevity and the chemical structure of compounds that may affect longevity.

Chemical molecular descriptors can be broadly categorized into three main groups, which describe a compound's chemical structure and its main properties. These groups are: hydrophobic, electronic and steric (size and/or shape) descriptors. Hydrophobicity descriptors describe the hydrophobic character of a chemical compound and how easily it can cross cell membranes, and they may also be important for receptor interactions. Electronic molecular descriptors describe the electron distribution in a chemical compound and its electrostatic interactions, therefore they give an indication of how strongly (in terms of affinity) and how specifically a chemical compound binds to specific receptors. Finally, steric descriptors describe the size and shape of the chemical compound. The size and shape of a compound may influence its binding with an enzyme or receptor binding sites and can also affect other psychochemical properties. Note that a chemical molecular descriptor can belong to more than one of the categories described above.

The top 20 selected features with the highest median variable importance are shown in Table 2. Considering just the top 20 features as shown in Table 2, there are slightly more GO terms (12 out of 20) than chemical molecular descriptors (8 out of 20). Those 12 GO terms include terms related to mitochondrial processes, terms related to enzymatic and immunological processes and terms related to metabolic and transport processes. Furthermore, the eight chemical molecular descriptors in the top 20 features contain descriptors related to electronic and steric (size and shape) effects, but not to hydrophobic effects directly.

**Table 2:** Top 20 selected features with highest median variable importance

Median Variable Importance	Feature	Feature type	Feature Description
14.4	a_nN	MD	Number of nitrogen atoms in the molecule
12.8	isomerase activity	GO	Catalysis of the geometric or structural changes within one molecule
11.8	macromitophagy	GO	Degradation of a mitochondrion by macroautophagy
11.6	macroautophagy	GO	Process in which cellular contents are degraded by lysosomes
11.1	protein disulfide isomerase activity	GO	Catalysis of the rearrangement of both intrachain and interchain disulfide bonds in proteins.
11.0	dipeptidase activity	GO	Catalysis of the hydrolysis of a dipeptide.
9.72	pyruvate metabolic process	GO	The chemical reactions and pathways involving pyruvate
9.47	PEOE_VSA+4	MD	Total positive van der waals surface area of atoms with atomic charge in the range of 0.20-0.25.
9.31	fatty acid transport	GO	The directed movement of fatty acids into, out of or within a cell, or between cells
8.79	mitochondrial electron transport, NADH to ubiquinone	GO	The transfer of electrons from NADH to ubiquinone mediated by the multisubunit enzyme known as complex I
8.64	vsurf_Wp2	MD	Polar volume at -0.5, a descriptor reflecting the polarizability of a molecule
8.57	isotype switching	GO	The switching of activated B cells from IgM biosynthesis to biosynthesis of other isotypes
8.40	translation	GO	The cellular metabolic process in which a protein is formed
8.18	Q_RPC-	MD	Relative negative partial charge, defined as the most negative atomic charge divided by the sum of all negative atomic charges in the molecule.
8.09	aerobic respiration	GO	The enzymatic release of energy from inorganic and organic compounds
7.98	a_IC	MD	Atom information content (total), defined as the entropy of the element distribution in the molecule multiplied by the number of atoms.
7.95	PEOE_VSA_FPPOS	MD	Fractional polar positive vdw surface area
7.86	triglyceride mobilization	GO	The release of triglycerides from storage within cells or tissues, making them available for metabolism.
7.79	chi1v	MD	Valence corrected molecular connectivity index (order 1)
7.70	bpol	MD	Sum of the absolute value of the difference between atomic polarizabilities of all bonded atoms in the molecule

GO: Gene ontology term; MD: Chemical Molecular descriptor

It can be seen from the list of important features that the vast majority of the most important features are very specific molecular and biological processes. However, these specific processes are generic in their applicability and occur across many tissues and organs. For example “isomerase activity” covers a broad range of various enzymes that catalyze reactions across many biological processes, such as in glycolysis and carbohydrate metabolism. Although it is evident that isomerase activity is relevant to metabolism (amongst other processes) and hence ageing, this feature is not specific enough to suggest practical targets for pharmacological intervention. In spite of this, some of the specific features have been linked with longevity and ageing processes.

GO terms related to metabolism encompass the vast majority of the GO term features listed in Table 2. These GO terms range from very general metabolism-related properties such as aerobic respiration to more specific processes such as dipeptidase activity, pyruvate metabolic process, fatty acid transport and mito-chondrial electron transport from NADH to ubiquinone. Given the involvement of metabolic factors in several theories of ageing such as the free radical theory of ageing, as well as the well-established effect of calorie-restriction on longevity, it is expectable that the compounds that affect ageing do so by interacting with these pathways and processes, as evidenced also by the importance of such features in the random forest model.

One apparent group of features that can be related to longevity and ageing are the GO terms related to autophagy (macroautophagy and macromitophagy) and mitochondrial processes. Macroautophagy is the process where cellular contents are degraded by lysosomes or vacuoles and recycled, and this process controls cytosolic protein and organelle degradation [27,28]. Whereas macromitophagy is the degradation of mitochondrion by macroautophagy and controls mitochondrial quality and quantity [29]. It is known that autophagy in general is associated with ageing processes. This can be evidenced by the occurrence of degenerative changes in mammalian tissues, similar to changes seen with ageing, as a result of genetic inhibition of autophagy. Moreover, pharmacological or genetic manipulations that increase life span in model organisms often stimulate autophagy. In the same way, there is a decrease in autophagy with increasing age in organisms, which leads to accumulation of damage [30] which is thought to be responsible for the functional loss in many biological and physiological processes as ageing occurs [31,32]. In addition to macroautophagy, mitophagy is specifically implicated in ageing. Mitophagy has been shown to be a selective, “non-random” process [33] that is governed by several biological pathways (see [34] for a review of the molecular mechanisms).

Mitochondrial respiration, and in particular electron transport chain, is the main source of reactive oxygen species. As a result, mitochondrial homeostasis is particularly affected by ageing, as ROS generation in mitochondria leads to mitochondrial protein and mtDNA damage [34]. Therefore, mitophagy can be regarded as a defense against oxidative stress, mitochondrial dysfunction, and ageing. This is supported by findings that along with mitochondrial biogenesis pathways, a key mediator of mitophagy and longevity assurance under conditions of stress in *C. elegans* (DCT-1) is upregulated when mitophagy is impaired [35]. It is therefore not unexpected to find in this work that chemical compounds that modulated mitophagy are also important promoters of longevity. It is interesting to note that in model organisms such as *C. elegans* disruption of mitochondrial electron transport chain processes can lead to increases in longevity, through genetic [36] or pharmacological interventions [37]. Finally, a related property, aerobic respiration, was also selected by the random forest model. Although aerobic respiration is a very broad term encompassing many processes that lead to the production of cellular energy, it is very well-associated with ageing through the known impact of mitochondrial function and caloric restriction.

Other GO features with links to longevity and ageing processes are protein disulfide isomerase activity and translation. Protein disulfide isomerase activity refers to the activity of isomerases that are involved in protein folding via formation and breakage of disulfide bonds within proteins in the endoplasmic reticulum (ER) [38,39]. The activity of this enzyme is key to protein folding and quality control in the ER. A number of studies have demonstrated that the levels of disulfide isomerase and their catalytic activity diminish with age [40]. Misfolding of proteins and ER stress are alleviated by the signalling pathway known as the ER stress response or the unfolded protein response, which involves protective measures to limit the protein load. These include up-regulation of ER chaperones involved in the refolding of proteins, activation of pathways leading to reduction of protein translation and degradation of misfolded proteins. Where ER stress cannot be reversed, cellular functions deteriorate and apoptosis will occur [41]. There is evidence in the literature to suggest that disruption of protein disulfide isomerase activity leads to ER stress and accumulation of misfolded proteins, which can give rise to age-related disease pathology [42]. Finally, the GO term translation has a clear biological relevance, since it is well-known that translation inhibition extends lifespan in *C. elegans* [43]. Translation has also been highlighted as a prime category in age-related genes in *C. elegans* in a recent paper by Fernandes et al. (2016) [44]. It is therefore evident that pathways involved in protein translation and folding may be a target of anti-ageing compounds, hence the significance of GO terms such as “translation” and “disulphide isomerase” in the random forest model.

The molecular descriptors in Table 2 indicate the molecular properties that impact the longevity effect of the compounds. From the eight molecular descriptors listed in the table, the majority are electrostatic descriptors such as PEOE_VSA+4, vsurf_Wp2, Q_RPC-, PEOE_VSA_FPPOS and bpol. These electrostatic parameters also carry information regarding the topology of the molecule, and along with steric parameters such as chi1v and a_IC explain the interaction and binding of the compounds with their target sites. These targets/processes are in addition to those already described in the model by the biological features (GO terms).

Overall, even though the used dataset (like any other biological dataset) is somewhat biased by the fact that some genes have been much more studied than others [44], some of the most important features shown in Table 2 can be related to important and known biological processes of ageing and longevity, such as those related to autophagy and mitochondrial processes. Furthermore, the other selected biological and chemical features are a good starting point that warrants further investigation, to further link the chemical and biological features of chemical compounds with longevity and underlying biological ageing processes.

### Predictions of novel potential life-extending compounds

The best model built from the DrugAge dataset (using GO terms and chemical descriptors) was used to predict the probability of the class “increase lifespan” for over 6,000 compounds from the DGIdb database v2 [45], where the class label of each compound is unknown. By using the predicted class probabilities we can rank and prioritise those compounds with the highest probability of increasing the lifespan of *C. elegans*. The list of all compounds predicted from the DGIdb dataset and their associated class probabilities can be found in the Supplemental Data, and the class probabilities for the top 20 compounds can be found in Table 3.

**Table 3:** Top 20 chemical compounds with the highest lifespan-increase class probability from the external screening dataset

Chemical Compound Name	Predicted Probability
acrolein	0.691
valspodar	0.683
ganirelix	0.674
acetaldehyde	0.669
mmk-1	0.667
rdp-58	0.665
cetrorelix	0.657
gal-b5	0.656
m40	0.654
DB03393	0.650
bortezomib	0.650
ro 25-1392	0.650
gv1001	0.650
lactose	0.650
ergotamine	0.650
cardiolipin	0.642
dactinomycin	0.642
abt-510	0.640
aplyronine a	0.637
valinomycin	0.637

As shown in Table 3 the highest predicted class probability for a compound in DGIdb was 0.69. Although not close to 1, this can be considered a relatively high probability, considering that the baseline probability (relative frequency) of the class “lifespan increase” in the DrugAge dataset used to build the model was only 0.20. In this section, we focus on the 50 “top hit” DGIdb compounds, with the highest values of probabilities for the predicted class “lifespan increase”. In general, the top hit compounds predicted to have longevity enhancing effects fall into four groups: compounds affecting mitochondria, compounds used in treatments for cancer, anti-inflammatories, and compounds used in gonadotropin-releasing hormone therapies.

#### Compounds related to mitochondrial processes

Acrolein (lifespan increase class probability = 0.69) was the top hit in our screening dataset. Acrolein is a highly reactive electrophile and a building block to many other chemical compounds, including the amino acid methionine. This compound has been shown to be an electron transport chain inhibitor, leading to mitochondrial dysfunction [46]. Acrolein is implicated in pathways such as p53 and the NF-κB inflammation pathway [47]. Acrolein is toxic at high concentrations [46], but at lower doses *in vitro* exposure to acrolein inhibits NF-κB activation, suggesting that inhibition of NF-κB gives rise to acrolein's anti-inflammatory properties – however, the evidence is conflicting [48,49]. Therefore, the high probability of lifespan increase predicted by our model, despite the known toxicity of acrolein, may result from the contribution of a large diversity of the pathways affected by this compound, some of which are desirable for longevity.

Other compounds affecting mitochondrial processes include valinomycin and cardiolipin (both with lifespan increase class probability = 0.64). Valinomycin is a potassium ionophore and causes mitochondrial dysfunction by uncoupling oxidative phosphorylation in the electron transport chain [50]. Cardiolipin is a dimeric phospholipid found in the inner mitochondrial membrane (IMM), where it plays a major role in oxidative phosphorylation. Alterations in the content and composition, and peroxidation of cardiolipin leads to mitochondrial dysfunction [51,52]. Decrease in cardiolipin content has been observed in ageing brain, and in several pathologies including myocardial ischemia, heart failure and Parkinson's disease [53]. Therefore, it is expectable that cardiolipin administra-tion is predicted to promote longevity.

#### Anti-cancer drugs and longevity

Anti-cancer compounds from our top 50 hits in the DGIdb dataset include drugs such as temsirolimus, valspodar and bortezomib. Interestingly, temsirolimus (lifespan increase class probability = 0.62) is a derivative and pro-drug of sirolimus – also known as rapamycin. Rapamycin was the first pharmacological compound shown to extend lifespan in both genders in mice models [54,55], *C. elegans* [56] and *D. melanogaster* [57]. Numerous studies indicate that inhibition of the TOR (Target of Rapamycin) kinase is implicated in lifespan control [58,59]. Temsirolimus also inhibits mTOR, and this compound has been shown to improve certain cellular phenotypes in accelerated ageing models via increasing autophagy [60].

Valspodar (lifespan increase probability = 0.68), the second top-hit in our screening dataset, is an experimental chemosensitizer drug. Valspodar desensitizes tumor cells making them more vulnerable to anti-cancer drugs, due to its ability to inhibit P-glycoprotein (P-gp), which is overexpressed in many cancer cells. However, possibly of more relevance is the apoptotic effect of valspodar (and its structurally related compound, cyclosporine A) that stems from their disruption of mitochondrial membrane potential leading to mitochondrial dysfunction [61].

Bortezomib (lifespan increase probability = 0.65) is a proteasome inhibitor, and studies have shown that the inhibition of proteasome activity by bortezomib is associated with enhanced apoptosis due to inhibition of NF-κB activity [62,63]. However, this compound also leads to the accumulation of misfolded proteins and ER stress followed by unfolded protein response (UPR) and macroautophagy [64], which may potentially lead to longevity promotion.

Dactinomycin (lifespan increase probability = 0.64) interferes with ribosome biogenesis through the inhibition of RNA polymerase I [65], which leads to the activation of p53 [66]. Inhibition of the mTOR pathway leads to a reduction of ribosome biogenesis and increases lifespan in several species [54,57,67]. mTOR and p53 signalling pathways are connected by a number of different mechanisms, highlighting a complex relationship [66,68,69]. Considering that there are similar signaling molecules involved in both cancer and ageing [70,71], such as mTOR [72], p53 [69] and NF-kB [73], it is not unexpected to find anti-cancer drugs in our list of top hit compounds. However, this could be due to research bias, where anti-cancer drugs may be overrepresented in datasets (including DrugAge) due to the extensive study of cancer therapies.

#### Chemical compounds with anti-inflammageing effects

Ageing has been characterized by chronic, low-grade inflammation, also labeled as “inflammageing” [74]. Human studies have shown that suppression of chronic inflammation is a major determinant of successful longevity, over a very wide age range up to extreme old age [75,76].

The compound rdp-58 (lifespan increase class probability = 0.67), tested for the treatment of the inflammatory disorder ulcerative colitis [77,78], leads to a reduction of proinflammatory (tumor necrosis factor alpha) TNF-α and interleukins (ILs) such as interferon-γ, IL-2, IL-6, and IL-12 [79].

Ergotamine (lifespan increase probability = 0.65), a vasoconstrictor used for the treatment of migraines, has also been shown to reduce the level of proinflammatory TNF-α [80]. Dihydroergotamine methanesulfonate increases longevity in *C.elegans* [18] and was used to build our models. Dihydroergotamine methanesulfonate is a derivative of ergotamine, so this can explain the predicted pro-longevity effects for ergotamine.

The compound ro 25-1392 (lifespan increase probability = 0.65) is a type II vasoactive intestinal peptide receptor (VIPR2) agonist [81]. ro 25-1392 is an analogue of vasoactive intestinal peptide (VIP), which binds to both VIPR1 and VIPR2, leading to protection in models of inflammatory and autoimmune conditions [82,83].

#### Reproductive hormone factors and longevity

Gonadotropin-releasing hormone (GnRH) is responsible for the release of follicle-stimulating hormone (FSH) and luteinizing hormone (LH) in the pituitary gland, promoting the production of testosterone and estrogen. It is a part of the hypothalamic–pituitary–gonadal axis, which helps in the regulation of reproductive and immune systems [84].

In our list of top hit compounds there are examples of GnRH antagonists, such as ganirelix [85] and cetrorelix [86] (lifespan increase class probabilities 0.67 and 0.66, respectively); and agonists such as nafarelin [87] and histrelin [88,89] (lifespan increase class probabilities 0.63 and 0.62, respectively). Both antagonists and agonists (whose continued use leads to desensitisation of GnRH receptors) of GnRH receptors lead to the reduction of FSH and LH.

The decline in GnRH has been shown to contribute to ageing-related changes such as bone fragility and reduced neurogenesis in mice. Zhang [90] showed in mice that activation of NF-κB in the hypothalamus led to a reduced production of GnRH by neurons and that continued activation led to accelerated ageing, whereas GnRH treatment reduced neurogenesis and decelerated ageing. These findings suggest a link between inflammation and ageing related to GnRH. However, whether this relationship involving GnRH applies to humans and primates is questionable, as it appears that female primates have higher levels of GnRH with increasing age [91], whereas in Norway rats GnRH levels decreased with increasing age [92]. It is therefore apparent that GnRH has some role in longevity independent of its role in reproduction.

## CONCLUSIONS

In this work we analysed data from the DrugAge database [10], which contains information about chemical compounds and their effect on the lifespan of organisms. We focused on compounds administered to *C. elegans*, since the majority of compounds in DrugAge have been evaluated in this model organism. For our data analysis, we used the machine learning method random forests, which builds a classification model to predict whether or not a chemical compound will increase the lifespan of *C. elegans*, based on predictive features describing that compound. We built three types of classification models, using either chemical descriptors or Gene Ontology terms, or both types of features. The dataset with both types of features led to the highest predictive accuracy in our experiments.

We used a score calculated by the random forest method to identify the most relevant features. Among the 20 highest score features, there are several GO terms which have a well-established association with the ageing process such as “macromitophagy” and “macroautophagy”. The high score of these GO terms is consistent with the fact that pharmacological or genetic interventions that increase lifespan in model organisms often stimulate autophagy [44]. Another example of a relevant GO term in the top 20 features was “translation”. It is well-known that translation inhibition extends lifespan in *C. elegans* [43]. The interpretation of the chemical features in the top 20 features is more difficult, since they refer to low-level chemical properties rather than broader biological processes – in general, those chemical features refer to electronic, size and shape effects of the compounds.

Furthermore, we applied the best classification model built by the random forest to a screening “external” dataset with compounds from the DGIdb database, where the effect of the compounds on an organism's lifespan is unknown. The predictions of that model were used to identify the “top hit” compounds in the DGIdb dataset, i.e. compounds with higher probabilities of increasing lifespan in *C. elegans*. We observed that these top hit compounds can be broadly divided into four groups: compounds affecting mitochondria, compounds for cancer treatment, anti-inflammatories, and compounds for gonadotropin-releasing hormone therapies.

In conclusion we have built, using machine learning, a model to predict the longevity effects of chemical compounds in *C.elegans*, using the recently published DrugAge dataset. The list of top-hit compounds and their analysis contributes to our knowledge of likely longevity-extending compounds, and experimental confirmation of these predictions would be an interesting direction for future research.

## METHODS

### Dataset creation

Chemical compounds that increased longevity in *C. elegans* were extracted from the DrugAge database (Build 2, release date: 01/09/2016) [10], available from the Human Ageing Genomic Resources website [9]. These compounds were assigned a positive class label (i.e. increased lifespan). Additionally, compounds that were found not to increase or had no effect on longevity in *C. elegans* were collected from the literature and were assigned a negative class label. The sets of positive and negative labelled compounds were combined to form the dataset for modelling. For ease, hereafter reference to the DrugAge dataset for modelling describes the positive entries from DrugAge plus the negative class label compounds. The number of positive and negative entries obtained were 229 and 1163 respectively, after dataset curation. The list of negative entries is present in the Supplemental Data. Compound entries from the DGIdb database v2 [45] were used to test and prioritise chemical compounds for longevity effects from the classification models built from the DrugAge dataset. The DGIdb dataset is used as our independent screening (or “external”) dataset, where the compounds’ longevity class labels are unknown.

#### Calculation of chemical molecular descriptors for the datasets used

For calculation of chemical molecular descriptors for chemical compounds, SMILES (Simplified Molecular-Input Line-Entry System) codes, which are line notations encoding the chemical structure, were extracted using PubChem [93] or ChemSpider (http://www.chemspider.com). For compounds where the chemical structure was not available, the structure was drawn directly from the literature reference (if available) and the SMILES code extracted. Compounds were removed at this stage if there was no SMILES code available, contained heavy inorganic metals, were duplicate compounds or were compound mixtures. Additionally, if there were chemical isomers with the same class label, only one entry was kept. For the DGIdb dataset, compounds were removed if they had a molecular weight greater than 3000 Daltons due to software restraints.

For molecular descriptor calculation, MOE (Chemical Computing Group Inc.) v2013.0.8 and Advanced Chemistry Development ACD Labs/LogD Suite v12 were used to calculate 2D and 3D molecular descriptors using the desalted and minimised chemical structure (using the semi-empirical method MOPAC PM6) of each compound. For the DrugAge dataset, molecular descriptors were removed if they had greater than 98% constant values, resulting in a final number of 268 molecular descriptors. The same 268 molecular descriptors were also calculated for the compounds in the DGIdb dataset. Due to software limitations, some molecular descriptors could not be calculated for some compounds. Using the “missForest” R package v1.4 [94], missing values were imputed for chemical molecular descriptors where this occurred, in both the DrugAge and the DGIdb datasets. A total of 1392 compound entries had molecular descriptors calculated for the DrugAge dataset (229 positive and 1163 negative entries). For the DGIdb dataset, 6802 entries had molecular descriptors calculated.

#### Computation of biological descriptors for the datasets used

Biological descriptors for each compound in each of the datasets were obtained by extracting drug-gene interactions using the DGIdb v2 database [45] and drug-protein interactions using the STITCH v4 database [95]. For drug-protein interactions using STITCH, only the top 100 interactions with a confidence score greater than 0.450 (considered a ‘medium confidence strength’ in STITCH) were used. The drug–gene/protein inter-actions obtained were annotated using GO terms (biological process, molecular function and cellular component terms) using the ClueGO plugin [96] in Cytoscape v3.3.0 [97]. For ClueGO, the parameters selected were “GO term fusion” and the entire “GO tree interval” using a background of *Homo sapiens* as the reference set. *Homo sapiens* annotations were used rather than *C. elegans* due to the poor representation of GO terms for this model organism. There were 10757 GO terms that were created as categorical biological features for the datasets. For each GO term, for each compound a categorical “yes” or “no” feature value was provided for each compound, indicating whether or not, respectively, the protein interacting with that compound was annotated with that GO term.

For this work, classification models were built using datasets with different combinations of chemical and biological descriptors (features) from the original DrugAge dataset. The different datasets used were: Firstly, a dataset using only biological descriptors (GO terms) as features. Secondly, a dataset using only chemical descriptors as features. Thirdly, a dataset using both biological and chemical descriptors as features. A summary of compound numbers for each of the different versions of the DrugAge dataset and the DGIdb dataset can be found in Table 4. Datasets DrugAge_1 and DrugAge_3 have fewer compounds than dataset DrugAge2 because they use GO terms as features, and compounds were discarded because their interacting proteins had no GO term annotation.

**Table 4:** Compound numbers for the DGIdb dataset and different combinations of DrugAge datasets using different combinations of chemical and biological descriptors used in this work

Dataset	n Positive	n Negative	n Total	Type of features used
DrugAge_1	190	783	973	GO terms ONLY
DrugAge_2	229	1163	1392	Chemical Descriptor ONLY
DrugAge_3	190	783	973	GO terms + Chemical Descriptors
DGIdb	-	-	6802	GO terms + Chemical Descriptors

Notation used in the table: n – number of compounds; Positive – increases longevity; Negative – no effect or decrease in longevity; Biological descriptors – GO terms (all three types); Chemical descriptors – molecular descriptors calculated from the chemical structure of compound entries using cheminformatics software.

### Random forests

In this work we used a random forest algorithm [98]. For our classification task, a random forest algorithm builds a classification model consisting of a set of decision trees, where each tree predicts a class label for each new compound. The predictions from all of the trees are then counted, and the class label assigned to a new compound is the label (positive or negative) with the highest number of votes from all of the decision trees in the forest.

Random forest training, including parameter optimization (explained in more detail below), was performed using the “mlr” R package (developer version 2.9) [99], which is a general machine learning interface that works as a wrapper for a plethora of learning algorithms available in distinct R packages. We have trained the random forests that the mlr package imported from the “ranger” R package [21].

After building a random forest model, a measure of variable importance can be computed in order to identify the most relevant input variables (features) for predicting the class variable. We used a permutation-based method for measuring variable importance. In order to evaluate the predictive power of a feature, for each tree in the forest, this method computes the predictive accuracy of that tree using two versions of the data: with random permutation of the values of the variable being evaluated, and without random permutation (i.e., using the original data). These differences of predictive accuracies are averaged over all trees in the random forest to give the feature's permutation-based importance value. In this work the variable importance values were computed using the Boruta R package [21,24] with the unscaled, unconditional permutation-based variable importance measure [100], performing the analysis on 100 permutation-based random forests (varying the random seed used to generate the permutations).

### Measuring predictive accuracy

We use the Area Under the Receiver Operating Characteristic Curve (AUC) as the predictive accuracy measure in our experiments. This is a popular measure of predictive accuracy in both machine learning and bioinformatics, and copes well with imbalanced class distributions (such as in our datasets). The AUC value varies from 0 to 1, with 1 indicating a perfect classifier, which would correctly classify every instance; 0.5 indicating a classifier that randomly guesses the class (positive or negative label) for each instance, and 0 indicating the worst possible classifier, which would systematically misclassify every instance.

### Nested cross-validation and random forest parameter optimization

To measure the predictive accuracy of the models developed, we used a nested cross-validation procedure. First, the DrugAge dataset was randomly divided into 10 non-overlapping folds with approximately the same number of compounds in each fold. The external cross-validation procedure performs 10 iterations of the classification algorithm (random forest), each time using one of the folds as the test set and the other 9 folds as the training set. In each of these 10 external cross-validation iterations, an internal 10-fold cross-validation procedure was applied to the training set. That is, the training set was randomly partitioned into 10 folds of approximately the same size, and 10 iterations were performed, using one of the training folds as a validation set and the other 9 training folds as the learning set from which a random forest model is built. Hence, in total 100 iterations were performed.

This nested cross-validation structure was used to perform parameter optimization in a strict way, using only the training set and not the test set in each external cross-validation iteration. This is important because parameter optimization is part of the training of a classification algorithm, and it has to be done using the training set only. The test set is reserved purely for measuring generalization ability, i.e. the ability to correctly predict the classes of compounds not observed during training.

A random forest algorithm has two major parameters which are often optimized for the target dataset, namely: the number of trees in the forest (ntrees) and the number of candidate features evaluated to select the best feature in each tree node (mtry) [101]. In order to optimize these parameters, we tested five settings for the ntrees parameter, namely ntrees = 100, 300, 500, 700, and 900; and three settings for the mtry parameter, namely: the square root of the number of features in the dataset (the default setting in [23,102]), as well as the half and the double of that default setting. For other parameters, their default values in the “mlr” R package were used.

In the above nested scheme, in each iteration of the external cross-validation procedure, parameters are optimized as follows: we ran the random forest algorithm 15 times, each time with a different combination of parameter settings (5 ntrees settings times 3 mtry settings), and each time performing an internal cross-validation in the training set. The parameter setting combination producing the best median AUC value across the 10 internal cross-validation iterations was chosen as the optimized parameter settings for the current external cross-validation iteration, and then a random forest algorithm with those optimized parameter settings was ran using the entire training set available at the current external iteration, with its predictive accuracy being evaluated on the test set for that iteration. The final measure of predictive accuracy reported in the Results section is the median AUC value across the 10 test sets in the external cross-validation procedure.

### Evaluation methodology

We evaluate the results of the random forest in three ways. First, we measure its predictive accuracy, using the well-known cross-validation procedure that is commonly used in supervised machine learning. Second, we identify the GO terms most relevant for predicting a compound's effect on *C. elegans*’ lifespan, according to a feature score calculated by the random forest, and discuss the relevance of such GO terms to the biology of ageing research. Third, we apply the best classification model built by the random forest to a screening “external” dataset with compounds from the DGIdb database, where the effect of the compounds on an organism's lifespan is unknown. That model's predictions are then used to identify the “top hit” compounds in the DGIdb dataset which have more potential as a pharmacological intervention against ageing in *C. elegans*.

## SUPPLEMENTARY MATERIAL TABLES


